# Hydrogen-Rich Water Ameliorates Autistic-Like Behavioral Abnormalities in Valproic Acid-Treated Adolescent Mice Offspring

**DOI:** 10.3389/fnbeh.2018.00170

**Published:** 2018-08-06

**Authors:** Qingjun Guo, Xi Yin, Meng Qiao, Yujiao Jia, Dandan Chen, Juan Shao, Tyler W. Lebaron, Yuan Gao, Haishui Shi, Bin Jia

**Affiliations:** ^1^Department of Surgery, Hebei Medical University, Shijiazhuang, China; ^2^Department of Functional Region of Diagnosis, Hebei Medical University Fourth Hospital, Hebei Medical University, Shijiazhuang, China; ^3^College of Basic Medicine, Hebei Medical University, Shijiazhuang, China; ^4^Department of Senile Disease, The Third Hospital of Hebei Medical University, Hebei Medical University, Shijiazhuang, China; ^5^Molecular Hydrogen Foundation, Kissimmee, FL, United States; ^6^Department of Biochemistry and Molecular Biology, Hebei Medical University, Shijiazhuang, China; ^7^Neuroscience Research Center, Hebei Medical University, Shijiazhuang, China; ^8^Hebei Key Laboratory of Forensic Medicine, Department of Forensic Medicine, Shijiazhuang, China; ^9^Collaborative Innovation Center of Forensic Medical Molecular Identification, Hebei Medicial University, Shijiazhuang, China; ^10^Lingshui General Hospital, Lingshui, China

**Keywords:** molecular hydrogen, autism, valproic acid, inflammation, interleukin 6, tumor necrosis factor-α

## Abstract

Due to its anti-inflammatory and anti-oxidative effects, recent research has demonstrated that molecular hydrogen can serve as a new medical approach for depression, anxiety and traumatic brain injury. However, its potential effects on neurodevelopmental diseases, such as autism are still elusive. The present study aims to investigate the potential effects of hydrogen-rich water (HRW) administration on valproic acid (VPA)-induced autistic-like behavioral deficits, and the associated underlying mechanism in adolescent mice offspring. Pregnant ICR mice were randomly divided into five groups (*n* = 6). One group was injected with saline (NAV group) and provided hydrogen-free water. The other four groups were injected with VPA (600 mg/kg, intraperitoneally, i.p.) on pregnant day (PND) 12.5. One group was provided with hydrogen-free water (VEH group) and the other three groups were provided HRW at different segments, postnatal day 1 (PND 1) to PND 21 (PHV group), PND 13 to PND 21 (PVS group) or from PND 13 to postnatal day 42 (PVL group). Behavioral tests, including open field, novelty suppressed feeding (NSF), hot plate, social interaction (SI) and contextual fear memory tests were conducted between postnatal day 35–42. We found that HRW administration significantly reversed the autistic-like behaviors induced by maternal VPA exposure in the adolescent offspring of both male and female adolescent offspring. Furthermore, HRW administration significantly reversed the alternation of serum levels of interleukin 6 (IL-6) and tumor necrosis factor-α (TNF-α), but without any effects on the BDNF levels in maternal VPA-exposed mice offspring. These data suggest the need for additional research on HRW as a potential preventive strategy for autism and related disorders.

**Lay Summary**: Maternal VPA injection induces autistic-like behavioral deficits in adolescent mice offspring. HRW administration ameliorates autistic-like behavioral deficits. HRW administration reverses the alternation of serum levels of IL-6 and TNF-α induced by VPA.

## Introduction

Autism spectrum disorder (ASD) is a neurodevelopmental disorder with persistent impairments of social interactions (SIs), communication deficits, restricted repetitive behavior and higher anxiety-like behaviors (Hollocks et al., 2014; Chahrour et al., 2016). Increasing scientific data links both peripheral and brain inflammation with the pathogenic development of autism. Those with autism exhibit signs of neuroinflammation, dysregulated inflammatory responses and immune abnormalities. These characteristics are observed in not only the perinatal period, but also throughout life (Masi et al., 2017; Meltzer and Van de Water, 2017; Prata et al., 2017; Varghese et al., 2017). Results from postmortem studies also showed that patients with ASD have neuroinflammation in certain areas of the brain, and further analyses suggest a strong immune response (Lee et al., 2017; Varghese et al., 2017; Courchesne et al., 2018). Additionally, autistic individuals can have an abnormal immune system, which would in turn increases their risk to chronic infection and autoimmune disorders (Lee et al., 2017). Recent studies in mice have demonstrated that maternal immune activation can significantly alter the behavior of the offspring, and thus decreased sociability and other autistic-like behaviors (Careaga et al., 2017; Lombardo et al., 2018). Moreover, the mice also have immune dysregulation, which corroborates the clinical evidence that links perinatal infection with autism. Anti-inflammatory strategies and antioxidants exert significant anti-autistic-like effects in animals and in individuals with autism (Bronson and Bale, 2014; Singh et al., 2014; Al-Amin et al., 2015).

Biomedical researchers have recently been interested in molecular hydrogen due to its anti-oxidative, anti-apoptotic and anti-inflammatory effects (Ichihara et al., 2015; Huang, 2016). Molecular hydrogen can be provided by inhalation of hydrogen gas, injection of hydrogen-rich saline (HRS) or oral intake of hydrogen-rich water (HRW). Molecular hydrogen can readily permeate through biomembranes such as the blood-brain barrier, blood-testis barrier and placental barrier, thus benefiting hard-to-reach organs (e.g., brain) and organelles due to its low molecular mass, non-ionic state and hydrophobic properties. An increasing number of studies report that molecular hydrogen offers important neuroprotective benefits in depression, anxiety, neuropathic pain, Parkinson’s disease, cognitive impairment and brain injury via attenuating excessive inflammatory response and oxidative stress (Imai et al., 2016; Zhang et al., 2016; Gao et al., 2017; Iketani and Ohsawa, 2017; Wen et al., 2017). In mice, HRW consumption *ad libitum* prevented cognitive impairment with an associated suppression of the markers of oxidative stress, malondialdehyde and 4-hydroxy-2-nonenal, and reversed the suppression on neural proliferation of the hippocampus caused by chronic physical restraint (Nagata et al., 2009). Similarly, HRW administration prevented cigarette smoke-induced pulmonary emphysema, suppressed anxiety and protected against excessive oxidative stress, alleviated ethanol-induced fatty liver, mitigated lipopolysaccharide-induced neuroinflammation and facilitated recovery of behavioral sickness in rodents (Lin et al., 2017; Masuda et al., 2017; Suzuki et al., 2017). Most recently, in CMS-treated mice (chronic mild stress) HRW exerted antidepressant-like effects by preventing oxidative stress, inflammation and apoptosis in the prefrontal cortex and hippocampus. Inhalation of molecular hydrogen could enhance resilience to acute and chronic stress in mice (Zhang et al., 2016; Gao et al., 2017). These results strongly suggest that molecular hydrogen administration may be a novel medical approach for many types of diseases, specifically those related to the central nervous system (Ohno et al., 2012).

Based on the dysregulation of the immune system of autism, and the anti-inflammatory characteristics of hydrogen, the present study aimed to investigate the potential effects of HRW on autistic-like behaviors using a maternal valproic acid (VPA)-exposed-mice model, and explore the associated mechanism focusing on the peripheral serum levels of interleukin 6 (IL-6) and tumor necrosis factor-α (TNF-α) assessed by Enzyme-Linked Immune-Sorbent Assay (ELISA) analysis.

## Materials and Methods

### Animal

Thirty female and 15 male ICR mice (20–25 g) were purchased from the Beijing Vital River Laboratory Animal Technology Co. Ltd., China. The rodents were kept in a climate-controlled environment, at a consistent temperature (22 ± 2°C), humidity (≈60%), a 12-h light/dark cycle (dark at 8:00 a.m.). Mice had *ad libitum* access to food and water. Each study followed the National Institutes of Health Guide for the Care and Use of Laboratory Animals guidelines. The investigative methods were approved by the Local Committee on Animal Care and Use and Protection of the Hebei Medical University.

### Hydrogen-Rich Water and Drug Administration

HRW (H_2_ concentration >1.8 mg/L, 245 mL) generously provided by Beijing Hydrovita Beverage Co. Limited (Beijing, China) and stowed at ambient pressure and temperature in an aluminum can with no head space. VPA (purchased from Sigma-Aldrich, Shanghai, China) was used following the procedure of previous reports to induce autistic-like behavior in mice (Al-Amin et al., 2015; Hara et al., 2017; Yamaguchi et al., 2017). VPA was dissolved in 0.9% NaCl solution, and the volume of injection was 10 mL/kg. To investigate the effects of hydrogen pretreatment and posttreatment on VPA-induced abnormalities, the pregnant mice were randomly divided into five groups (*n* = 6 per group). One group of mice (NAV group) was injected with saline on the 12.5th day of pregnancy (GD12.5) and provided with hydrogen-free water for the duration of the study. The other four groups of mice were injected intraperitoneally with 600 mg/kg of VPA on GD12.5. One group was provided with hydrogen-free water (VEH group) for the duration of the study. The other three groups were provided HRW from GD 1 to postnatal day 1 (PND1; PHV group), from GD 13 to PND 21 (PVS group) and from GD 13 to PND 42 (PVL group). All mice offspring were weaned at PND 21 and kept normal rearing for 4–6 per cage.

### Open Field Test

The open field test (OFT) was carried out based on previous reports (Wu et al., 2016; Gao et al., 2017). The apparatus involved a (40 cm × 40 cm × 35 cm) square arena. Each mouse was put in the center of the apparatus. The test session was videotaped and analyzed by a video tracking system (SMART 3.0, Panlab, Spain). The time spent in the central zone, and total distance of mice during the 5-min test process was recorded to reflect the anxiety-like behaviors, and the horizontal locomotion activity, respectively.

### Novelty Suppressed Feeding

The novelty-suppressed feeding (NSF) test was done according to our recent studies (Gao et al., 2017; Gong et al., 2017). The apparatus was a (40 cm × 40 cm × 35 cm) square area. Before the test, all mice were deprived of food for 24 h. Each mouse was placed in the corner of the apparatus with a small pellet of food on the center of the floor. Mice would be taken away from the apparatus once the mice ate the food and were transferred to their home cages. The latency to feeding (in seconds, maximum time, 300 s), and food consumption during the 10 min in home cage were measured to assess the anxiety-like behaviors and the appetite of the mice.

### Hot-Plate Test

The hot-plate test (HPT, Hot/Cold Plate Model-35100-001, UGO Basile, Italy) was used to assess nociceptive responding at a fixed temperature (55 ± 1°C) according to the methods described previously (Al-Amin et al., 2015; Ansari et al., 2017). Briefly, the test-animals were set on the hot plate and the latency to respond was noted from the time between the placement of the mice on the plate and the licking of either hind paws.

### Social Interaction Test

The social interaction test (SIT) was conducted as described previously (Lucchina and Depino, 2014; Kazlauskas et al., 2016). The apparatus was made of black plastic boxes (69 cm × 22 cm × 25 cm) and consisted of three-chambers with two side compartments, which allowed mice to pass freely by an opening door and get to the center area. A cylinder iron case which could hold a mouse was placed on the center of each side chamber. The mice were placed in the main compartment and allowed to survey the area for 5 min (habituation). Then, an unfamiliar mouse (mouse strain, age and sex are same to the test one), acting as social stimulus, was placed in one of the cylinders at the other end with an empty iron cage. SI was observed during a 10-min period. The social side was randomly determined. The time of sniffing to the iron cage was recorded to estimate the social preference.

### Contextual Fear Memory

Contextual fear conditioning (CFC) study occurred in chambers of Plexiglas (30.5 × 30.5 × 43.5 cm). Each compartment was capable of delivering an electric shock through the stainless-steel rod floor. The chamber also consisted of an upper control panel with a video camera. According to our and other previous studies (Kim et al., 2016; Gong et al., 2017), prior the CFC training, a mouse was placed into the conditioning chamber to survey the area for 5 min. The mouse was then immediately returned to its colony room. The following day the same procedure was repeated. On the third day, the CFC training session was performed by placing a mouse in the same conditioning chamber for 2 min, followed by a 1 s foot shock (1 s, 0.6 mA). The foot shock reoccurred two more times with same intervals of 60 s. After the last foot shock, the mouse remained in the chamber for another 60 s before being taken out. The “freezing” behavior, defined as the absence of all non-respiratory movement (immobile for 1 s at least) was recorded. After behavioral testing of each mouse, the area was washed with 70% ethanol. Statistical analysis was determined by computer software (Shanghai Xinruan Information Technology Co. Ltd., Shanghai, China).

### Enzyme-Linked Immune-Sorbent Assay (ELISA)

ELISA was conducted as previously described (Wu et al., 2016; Gao et al., 2017). In brief, Blood sample of mice was collected through extracting eyeballs. Blood sample were placed at ambient temperature for 20 min and centrifuged at 2000 rpm for 20 min. The serum was transferred into new tubes for ELISA analyses. The measurement of serum levels of IL-6 and TNF-α were conducted with commercially available ELISA kits (IL-6 ml002293; TNF-α, ml002095; mlbio, China).

### Data Analyses

Data are expressed as the mean ± SEM. One-way analysis of variance (One-way ANOVA) was provided for the statistical analyses of behavioral testing and ELISA data of the VPA-exposed mice offspring. Bonferroni’s *post hoc* test was followed to determine the differences between groups. Student’s *t*-test was employed to compare two separate groups. Values of *P* < 0.05 were considered statistically significant (SPSS, v. 16.0, Chicago, IL, USA).

## Results

### HRW Administration Reversed the Anxiety-Like Behaviors in VPA-Exposed Mice Offspring

**Figure 1:**
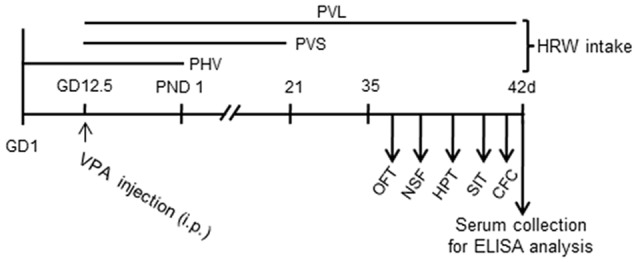
Experiment design. Mice were injected with valproic acid (VPA) (600 mg/kg, i.p.) on pregnant day 12.5 (GD12.5) and were provided with hydrogen-rich water (HRW) during pregnant phase and after postnatal day (PND). Behavioral tests of adolescent mice offspring were conducted from PND 35 to 42. Blood samples were collected after behavioral tests for Enzyme-Linked Immune-Sorbent Assay (ELISA) assays. OFT, open field test; NSF, novelty suppressed feeding; HPT, hot plate test; SIT, social interaction test; CFC, contextual fear conditioning test.

**Figure 2:**
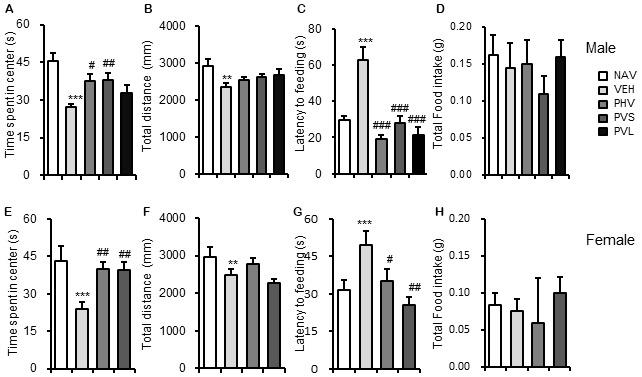
HRW administration reversed the anxiety-like behaviors in maternal VPA-exposed mice offspring. Anxiety-like behaviors were assessed by the OFT and NSF test. HRW administration significantly reversed the decreased time spent in the center of both male **(A)** and female **(E)** mice offspring, while had no significant effect on the total distance **(B,F)** in the OFT. HRW administration significantly reversed the increased latency to feeding of both male **(C)** and female **(G)** mice offspring, while had no significant effect on the total food intake **(D,H)** in the NSF test. OFT, open field test; NSF, novelty suppressed feeding test. Data are expressed as the mean ± SEM. **P < 0.01, ***P < 0.005 compared with the saline-injected group (NAV); ^#^*P* < 0.05, ^##^*P* < 0.01, ^###^*P* < 0.005 compared with the VPA-exposed group (VEH; *n* = 6–10 per group).

The VPA injection, HWR administration and all experimental procedures were conducted following the timeline indicated in Figure 1. We tested the effects of HRW on anxiety-like behaviors in VPA-exposed mice offspring. As shown in Figure 2, maternal exposure to VPA produced a significant increase in anxiety-like behaviors, as shown by decreased time spent in the central zone in the OFT (both *P* < 0.001, Figures 2A,E), increased latency to feeding in the NSF test (Figures 2C,G, *P* < 0.005, *P* < 0.001 for male and female, respectively). One-way ANOVA of the OFT data showed a significant effect of HWR administration for male (*F*_(3,36)_ = 3.548, *P* < 0.05) and female (*F*_(2,24)_ = 7.637, *P* < 0.005) mice offspring. *Post hoc* analysis showed that mice in PHV (both *P* < 0.05 for male and female, respectively) and PVS (both *P* < 0.01 for male and female, respectively) groups spent significantly more time in the central zone compared with that of VPA-exposed mice offspring. No significant effects of HRW administration occurred for total distance in the OFT (*F*_(3,36)_ = 1.323, *P* = 0.283; *F*_(2,24)_ = 2.775, *P* = 0.084 for male and female, respectively, Figures 2B,F). One-way ANOVA of the NSF data showed a significant effect of HRW administration for male (*F*_(3,36)_ = 16.513, *P* < 0.005) and female (*F*_(2,24)_ = 8.283, *P* < 0.005) mice offspring. *Post hoc* analysis showed that HRW administration in the PHV (*P* < 0.001 for male and *P* < 0.05 for female respectively), PVS (*P* < 0.001 for male and *P* < 0.001 for female) and PVL (*P* < 0.001 for male) significantly decreased the latency to feeding compared with that of VPA-exposed mice offspring. No significant effects of HRW administration occurred for total food intake of mice (*F*_(3,36)_ = 0.751, *P* = 0.53 and *F*_(2,24)_ = 0.476, *P* = 0.628 for male and female respectively, Figures 2D,H). These results indicate that maternal consumption of HRW reduces anxiety-like behaviors in VPA-exposed mice offspring.

### HRW Administration Reversed the Hyperpathia in VPA-Exposed Mice Offspring

**Figure 3:**
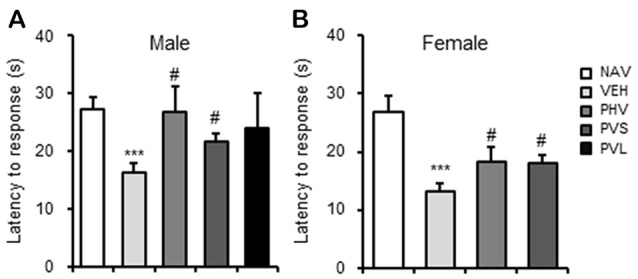
HRW administration reversed the hyperpathia in VPA-exposed mice offspring. HRW administration significantly reversed the maternal VPA exposure-induced decrease of the latency to response of both male **(A)** and female **(B)** mice offspring in the HPT. ***P < 0.005 compared with the saline-injected group (NAV); ^#^*P* < 0.05 compared with the VPA-exposed group (VEH; *n* = 6–10 per group).

Next, the potential effects of HRW administration on VPA-induced hyperpathia were assessed. As shown in Figure 3, Maternal exposure to VPA significantly decreased the latency to response (*P* < 0.001). One-way ANOVA of the HPT data showed a significant effect of HWR administration for male (*F*_(3,36)_ = 3.522, *P* < 0.05; Figure 3A) and female (*F*_(2,22)_ = 3.3, *P* < 0.05; Figure 3B) mice offspring. *Post hoc* analysis showed that the decreased latency to response of mice induced by VPA exposure could be significantly blocked by the PHV (both *P* < 0.05) and PVS (both *P* < 0.05) HRW administration protocols in both male and female offspring.

### HRW Administration Rescued the Impaired Social Preference in VPA-Exposed Mice Offspring

**Figure 4:**
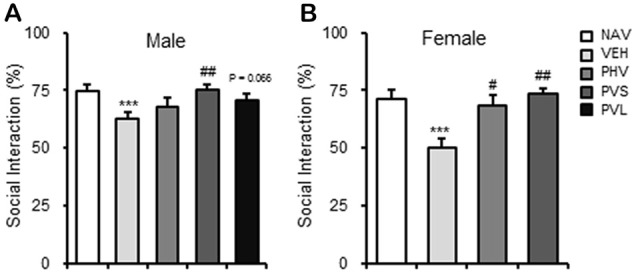
HRW administration rescued the impaired social behavior in VPA-exposed mice offspring. HRW administration significantly reversed the maternal VPA exposure-induced decrease of the SI index of both male **(A)** and female **(B)** mice offspring in the SIT. ***P < 0.005 compared with the saline-injected group (NAV); ^#^*P* < 0.05, ^##^*P* < 0.01 compared with the VPA-exposed group (*n* = 6–10 per group).

As shown in Figure 4, maternal exposure to VPA significantly impaired the social preference, which are reflected by the decrease of SI of both sexes of mice offspring (both *P* < 0.001). One-way ANOVA of the SIT data showed a significant effect of HWR administration for male (*F*_(3,28)_ = 3.33, *P* < 0.05; Figure 4A) and female (*F*_(2,25)_ = 13.019, *P* < 0.005; Figure 4B) mice offspring. *Post hoc* analysis showed that HRW administration in the PHV group significantly reversed the behavioral alteration induced by maternal VPA exposure in male mice offspring (*P* < 0.01). HRW administration in both PHV and PVS groups, reversed the decrease of SI in VPA-exposed female mice offspring (*P* < 0.05, *P* < 0.005, respectively).

### HRW Administration Had No Significant Effects on Memory Impairment in VPA-Exposed Mice Offspring

**Figure 5:**
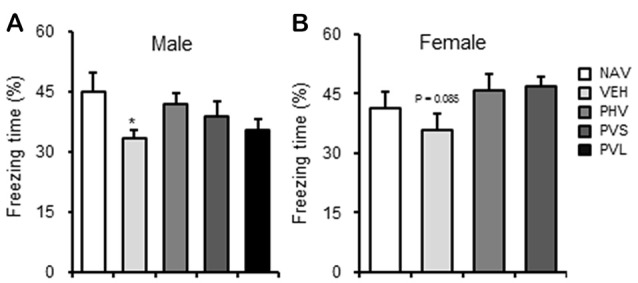
HRW administration reversed the memory impairment in VPA-exposed male mice offspring. HRW administration significantly reversed the maternal VPA exposure-induced the memory impairment of male **(A)** mice offspring and significantly enhanced that of female **(B)** mice offspring in the contextual fear memory test. *P < 0.05 compared with the saline-injected group (NAV; *n* = 6–10 per group).

As shown in Figure 5, VPA exposure induced a significant memory impairment of the male mice offspring in the CFC test (*P* < 0.05), with only a slight tendency of a decrease for female mice offspring (*P* = 0.085). One-way ANOVA of the CFC test data showed no significant effect of HWR administration for male (*F*_(3,35)_ = 1.881, *P* > 0.05; Figure 5A) and female (*F*_(2,22)_ = 2.472, *P* > 0.05; Figure 5B) mice offspring.

### HRW Administration Blocked the Inflammatory Response in VPA-Exposed Mice Offspring

**Figure 6:**
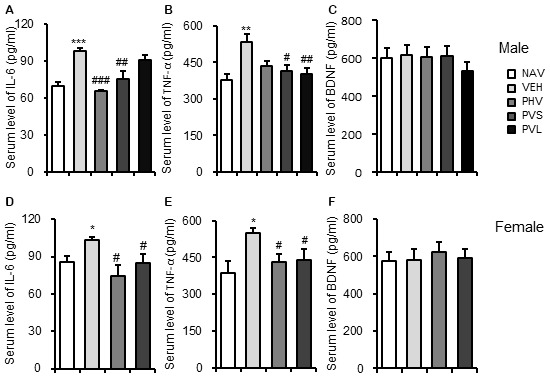
HRW administration blocked the inflammatory response in VPA-exposed mice offspring. ELISA data showed that HRW administration significantly reversed the increased serum levels of interleukin 6 (IL-6; **A,D**) and tumor necrosis factor-α (TNF-α) **(B,E)**, but not BDNF **(C,F)** of both male and female mice offspring. *P < 0.05, **P < 0.01, ***P < 0.005 compared with the saline-injected group (NAV); ^#^*P* < 0.05, ^##^*P* < 0.01, ^###^*P* < 0.005 compared with the VPA-exposed group (VEH; *n* = 6–10 per group).

To elucidate the mechanism mediating the anti-autistic effects of HRW, ELISA was carried out to detect the serum levels of IL-6, TNF-α and BDNF. As shown in Figure 6, VPA exposure significantly increased the serum levels of IL-6 (*P* < 0.01, *P* < 0.05 for Figures 6A,D, respectively) and TNF-α (*P* < 0.01, *P* < 0.05 for Figures 6B,E, respectively), but without significant effects on serum level of BDNF (both *P* > 0.05; Figures 6C,F) of both male and female mice offspring. One-way ANOVA of the IL-6 data showed a significant effect of HRW administration on male (*F*_(3,17)_ = 12.553, *P* < 0.001; Figure 6A) and female (*F*_(2,13)_ = 6.657, *P* < 0.05; Figure 6D) mice offspring. *Post hoc* analysis showed that HRW administration in both the PHV and PVS groups significantly reduced the increase of serum level IL-6 in male (both *P* < 0.01) and female (both *P* < 0.05) mice offspring. One-way ANOVA of the TNF-α data revealed a significant effect of HRW administration on male (*F*_(3,17)_ = 12.553, *P* < 0.001; Figure 6B) and female (*F*_(2,13)_ = 4.742, *P* < 0.05; Figure 6E) mice offspring. *Post hoc* analysis showed that HRW administration in PVS and PVL groups significantly attenuated the increased serum levels of TNF-α of male (*P* < 0.05, *P* < 0.01, respectively), and HRW administration in the PHV and PVS group also blocked the VPA-induced elevation of serum TNF-α (both groups *P* < 0.05) in the female mice offspring.

## Discussion

The present investigations corroborate previous reports, which demonstrate that maternal exposure to VPA has long-term negative effects on postnatal behaviors in mice offspring. Both pre- (PHV) and post (PVS, PVL)-administration of HRW reversed the VPA exposure-induced behavioral abnormalities, including the higher anxiety level, the altered pain sensation, the decrease of social behaviors and the impaired memory. Moreover, we found that HRW administration attenuates the increase of peripheral inflammation, assessed by increased serum levels of IL-6 and TNF-α in both male and female VPA-exposed mice offspring.

Molecular hydrogen is the smallest molecule, and has recently gained attention as a novel antioxidant-like molecule in preventive and therapeutic applications (Ohno et al., 2012; Ohta, 2014). Increasing evidence shows that molecular hydrogen administration provides many neuroprotective effects in central nervous system related diseases, such as ischemia-reperfusion injury, cerebral infarction, neonatal brain damage, radiation-induced damage, traumatic brain injury, cognitive impairments and Parkinson’s disease (Ohta, 2015; Huang, 2016). Most recently, our group and other groups’ studies showed that molecular hydrogen exerts neuroprotective effects on stress or drug dependance-induced memory impairment, depressive- and anxiety-like behaviors in mice, which are related to its efficient anti-oxidative, anti-inflammatory and anti-apoptotic activities (Ohno et al., 2012; Gao et al., 2017; Wen et al., 2017).

Maternal VPA-exposed rodent models are widely used to investigate the pathological mechanism of autism and to evaluate the anti-autistic efficiency of potential drugs and new strategies (Christianson et al., 1994; Martin and Manzoni, 2014). In animal, a single VPA administration in the prenatal stage results in morphological, behavioral and pathophysiological alterations, including higher anxiety status, memory impairment, deficits in SI and nociceptive alternation in the postnatal offspring (Al-Amin et al., 2015; Nicolini and Fahnestock, 2018). Consistent with previous reports, our present results showed that maternal VPA exposure induced higher anxiety-like behaviors, reflected by a decrease of time spent in center in the OFT, and a longer time to feeding in the NSF test in both male and female adolescent mice offspring. Meanwhile, VPA exposure induced lower locomotion activities in female mice offspring. Not only pre-administration (PHV), but also post-administration of HRW (PVS, PVL) significantly reversed VPA-induced anxiety-like behaviors in both sexes of mice offspring. Similar neuroprotective effects of HRW administration on the abnormal social behaviors and impaired memory ability in VPA-exposed mice offspring were found. For the VPA-induced hyperpathia behaviors, we found that maternal VPA exposure induced a decrease of latency to response of both male and female offspring during the HPT, which could be significantly reversed by HRW administration. These behavioral results highlight the significant neuroprotective effects of molecular hydrogen on VPA-induced autistic behaviors.

Significant evidence over the years suggests a pathophysiological relationship between cytokine alterations from abnormalities of the immune system and ASD (Jyonouchi et al., 2001; Ashwood et al., 2006). Dysregulated cytokine activities may assist the diagnosis of ASD subtypes that have similar characteristics and profiles, and also provide biological markers that assist in evaluating the benefits of different treatments during clinical trials. The pro-inflammatory cytokine IL-6, has been recognized as a cytokine that the brain acknowledges as a bio-signal of sickness. Elevated levels of IL-6 in ASD, both centrally and peripherally, is often reported (Ross et al., 2013). In mice, increased levels of this cytokine in the brain can promote autism-like behaviors via impairing synapse formation, development of the dendritic spine, and balance of the neuronal circuit. TNF-α is a primary modulator of inflammation and is increased in the cerebrospinal fluid of children with ASD (Al-Ayadhi, 2005; Masi et al., 2017). Peripheral IL-6 and TNF-α are also elevated in individuals with ASD compared to healthy controls (Masi et al., 2015). Our current findings are in line with these observations, showing peripheral elevations of IL-6 and TNF-α in the serum of maternal VPA-exposed mice offspring. These VPA-induced levels could be significantly reversed by ingestion of HRW. Besides the dysregulation of the immune system, elevated serum levels of BDNF were reported to be associated with autism (Qin et al., 2016; Meng et al., 2017). However, our study reported that serum levels of BDNF were not significantly changed in neither male nor female VPA-exposed mice offspring.

Additionally, although not measured in this study, HRW may benefit autism in three other ways. (1) Oxidative stress with accompanying altered levels of glutathione, superoxide dismutase, catalase and other cytoprotective enzymes are correlated with and implicated in the pathogenesis of autism (Chauhan and Chauhan, 2006). Although, not measured in our study, molecular hydrogen has been shown in many other studies to improve the redox status of the cell by decreasing oxidative stress, activating the Nrf2 pathway and regulating those endogenous antioxidants (Slezák et al., 2016). It has thus been suggested that HRW may be useful in the treatment and prevention of autism (Ghanizadeh, 2012). (2) It has been reported that the neuroprotective hormone ghrelin is lower in those with autism, and may be responsible in its pathogenesis (Al-Zaid et al., 2014). HRW can induce gastric ghrelin secretion, which mediated the neuroprotective effects of HRW in Parkinson’s disease (Matsumoto et al., 2013). The beneficial effects of HRW on Parkinson’s disease have been confirmed in clinical studies (Yoritaka et al., 2013). (3) A meta-analysis confirmed mitochondrial dysfunction is associated with autism, and various mitochondrial treatments could be beneficial in humans (Rossignol and Frye, 2012). Molecular hydrogen has been shown to improve the function of the mitochondria and promote mitochondrial biogenesis making it useful for those with mitochondrial myopathies, as shown in a clinical trial (Ohta, 2014). Considering the role of sodium/hydrogen exchanger in inflammatory-related brain diseases and the anti-inflammatory and antioxidant effects of molecular hydrogen (Cox et al., 1997; Shi et al., 2011), more research is warranted to better understand the underlying mechanism of HRW protecting against ASD.

To our knowledge these present findings are the first to show that HRW administration can significantly attenuate the autistic-like behavioral abnormalities, and dysregulation of the peripheral immune system induced by maternal VPA exposure. However, the precise mechanisms mediating these benefits of molecular hydrogen at mitigating the autistic-like behavior induced by VPA need to be further investigated. Considering the significant efficiency with no reports of toxic effects, the high safety and simplicity of use, HRW should be further studied as a potential novel method for preventing and treating ASD in the future.

## Author Contributions

HS and BJ conceived and designed the experiments. QG, MQ, YJ and DC performed the behavioral tests. XY, JS and YG analyzed the behavioral data and prepared the figures. QG, XY and JS conducted the ELISA assays and analyzed the data. HS, BJ and TL wrote and/or revised the article.

## Conflict of Interest Statement

The authors declare that the research was conducted in the absence of any commercial or financial relationships that could be construed as a potential conflict of interest.
